# Clinical and genomic characterization of *Staphylococcus argenteus* isolates from a tertiary hospital in Beijing

**DOI:** 10.3389/fmicb.2026.1876812

**Published:** 2026-07-28

**Authors:** Dequan Tian, Yunyi Liu, Ying Li, Diandian Chen, Leyang Ju, He Lan, Wenshuo Yang, Xiaoyi Zhao, Peichang Wang, Jingrong Cao

**Affiliations:** 1 Department of Clinical Laboratory, Xuanwu Hospital Capital Medical University, National Gerontic Disease Clinical Research Center, Beijing, China; 2 Department of Clinical Laboratory, Xiongan Xuanwu Hospital, Xiong’an New Area, Hebei, China

**Keywords:** antimicrobial resistance, biofilm formation, emerging pathogen, MALDI-TOF MS, multilocus sequence typing (MLST), *Staphylococcus argenteus*, virulence factors, whole-genome sequencing (WGS)

## Abstract

**Background:**

*Staphylococcus argenteus*, an emerging member of the *Staphylococcus aureus* complex (SAC) linked to severe infections globally, remains understudied in temperate regions such as northern China.

**Methods:**

In this single-center retrospective study, we re-identified 2,080 clinical isolates, which were initially classified as *S. aureus* using conventional phenotypic methods at a tertiary hospital in Beijing, China. Re-identification was performed using matrix-assisted laser desorption/ionization time-of-flight mass spectrometry (MALDI-TOF MS), followed by definitive confirmation of *S. argenteus* strains via *rpoB* and nonribosomal peptide synthetase (*NRPS*) gene sequencing and whole-genome sequencing (WGS). Phenotypic and genomic analyses were subsequently conducted, including the exploration of candidate MS spectral peaks, antimicrobial susceptibility testing (AST), *in vitro* biofilm formation capacity assessment, as well as resistome and virulome characteristics.

**Results:**

A total of three *S. argenteus* strains (0.14%) were confirmed among the SAC isolates. Four distinct MALDI-TOF MS spectral peaks (3452.7, 5004.2, 5303.8, and 6903.2 Da) were identified that may serve as potential discriminatory markers for *S. argenteus*, warranting validation in larger, geographically diverse multicenter cohorts. A conserved core virulome (adhesins, hemolysins, and immune evasion factors) was detected across all strains; one isolate uniquely harbored Panton-Valentine leukocidin (PVL, *lukS/F-PV*) and enterotoxin genes (*seg*, *sei*). All three isolates were susceptible to oxacillin, yet they exhibited variable biofilm formation capacities; two isolates demonstrated strong biofilm production, which may warrant further investigation as a potential contributor to localized persistence, though this remains a hypothesis requiring dedicated clinical and functional validation. Notably, despite the presence of genotypic resistance determinants, no fluoroquinolone resistance phenotypes were detected via *in vitro* AST.

**Conclusion:**

Our findings provide preliminary insights into the pathogenic potential of *S. argenteus* in this specific clinical environment. Although our large-scale screening revealed a remarkably low prevalence of *S. argenteus* in this local cohort, the small number of positive isolates highlights the need for multicenter surveillance to better understand its clinical impact and generalizability.

## Introduction

*Staphylococcus aureus* is a leading opportunistic human pathogen, causing infections ranging from mild skin and soft tissue infections to life-threatening bacteremia and sepsis (Cheung et al., 2021). In 2015, *Staphylococcus argenteus*, named for its characteristic silvery colony color, was formally recognized as a distinct species within the *Staphylococcus aureus* complex (SAC), alongside *S. schweitzeri* (Tong et al., 2015). While *S. argenteus* shares phenotypic and clinical traits with *S. aureus*, including coagulase positivity and the ability to cause severe infections, it exhibits unique genomic and phylogenetic characteristics (Fang et al., 2024; Yan et al., 2025).

**FIGURE 1:**
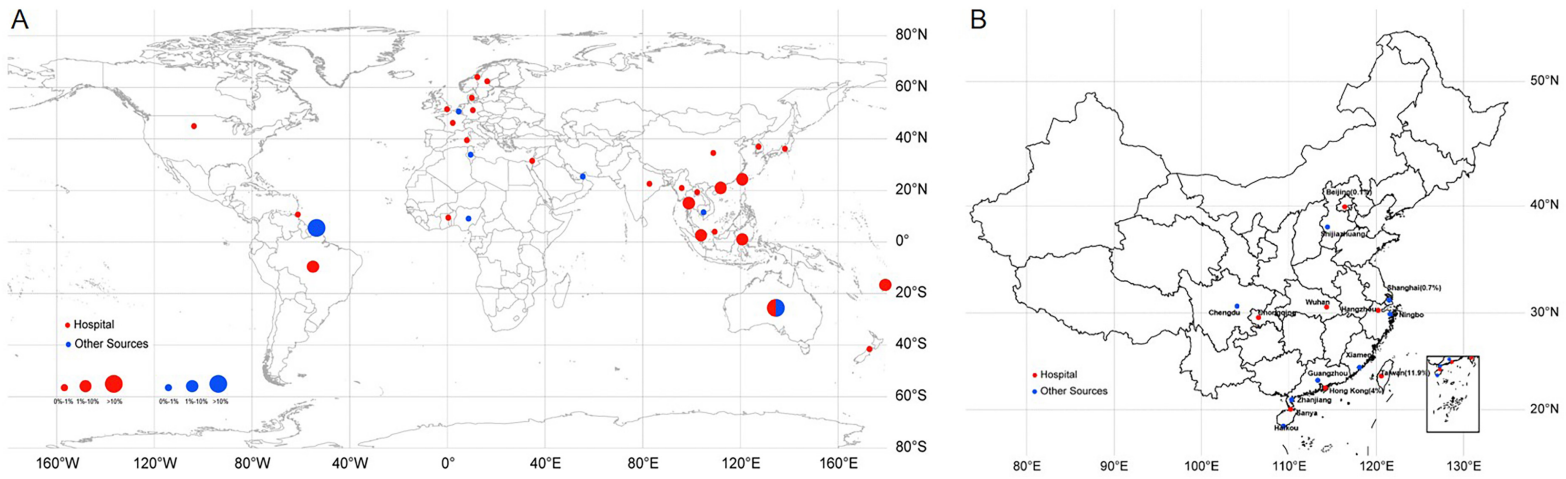
Geographical distribution and isolation rates of *S. argenteus*. **(A)** The global distribution demonstrates markedly higher isolation rates in tropical and subtropical regions compared to those in temperate zones. Red dots indicate strains originating from hospitals, while blue dots represent other sources (e.g., community and food). Dot size reflects the isolation rate, with larger dots indicating higher rates. **(B)** The regional distribution within China illustrates a higher prevalence in the southern and eastern coastal areas compared to that in the northern and inland regions.

Although global surveillance indicates that *S. argenteus* exhibits distinct geographic clustering, available research remains highly regionalized, thereby obscuring a comprehensive understanding of its global clinical burden and transmission dynamics. It has been detected across six continents ( > 20 countries, Figure 1A), with higher isolation rates in tropical and subtropical regions (8%–20%) (e.g., Australia, Southeast Asia, southern China) compared to < 1% in temperate areas (e.g., Europe, northern China) (Thaipadungpanit et al., 2015; Argudín et al., 2016; Chantratita et al., 2016; Supriadi et al., 2024; Aung et al., 2025; Tian et al., 2025). In China, it is more prevalent in southern and eastern coastal regions (such as Shanghai, Hangzhou, Guangdong, Taiwan, and Hong Kong) than in northern and inland areas (including Beijing, Chongqing, and Wuhan) (Chen et al., 2018, 2023; Figure 1B). This uneven distribution underscores the need for localized surveillance to characterize the clinical and molecular features of *S. argenteus* in low-prevalence regions.

Accurate identification of *S. argenteus* is challenging due to its close phylogenetic relationship with *S. aureus*. 16S rRNA gene sequencing fails to distinguish the two species ( > 99.8% sequence homology) (Tong et al., 2015), while housekeeping gene sequencing (e.g., *gap, rpoB, sodA, tuf*, and *hsp60*) improves resolution but is limited by cost and time constraints in routine clinical laboratories (Ng et al., 2009). MALDI-TOF MS offers rapid, cost-effective identification, yet its accuracy is currently hindered by insufficient *S. argenteus* spectra in reference databases (Eshaghi et al., 2021).

To address these diagnostic challenges and provide valuable local baseline data, we conducted an integrated phenotypic and genomic characterization of three rare clinical *S. argenteus* isolates retrospectively identified from a large cohort (*n* = 2,080) at a Beijing tertiary hospital. This study aimed to (1) explore candidate markers for MALDI-TOF MS-based identification; (2) delineate antimicrobial susceptibility profiles alongside underlying resistome characteristics and virulence gene repertoires; and (3) assess *in vitro* biofilm-forming capacity. These preliminary findings will enhance our understanding of *S. argenteus* as an emerging pathogen and provide a highly detailed exploratory framework for future large-scale research in temperate regions.

## Materials and methods

### Isolate collection and identification

A total of 2,080 clinical isolates initially identified as *S. aureus* using conventional methods (catalase/coagulase tests, VITEK 2 GP card) were collected from Xuanwu Hospital (January 2014 to December 2025). Isolates were stored at −80° C and subcultured on blood agar at 35° C for 24 h prior to analysis. MALDI-TOF MS identification was performed via Bruker Microflex LT system (Bruker Daltonics, Bremen, Germany) and Zybio EXS3000 system (Zybio Inc., Chongqing, China) following manufacturer protocols. Isolates with MALDI-TOF MS scores ≥ 2.0 for *S. argenteus* were validated by Sanger sequencing of the *rpoB* and *NRPS* genes (Zhang et al., 2016). Subsequently, the clinical data associated with these confirmed isolates were reviewed. The retrospective study was approved by the Ethics Committee of Xuanwu Hospital (Approval No.: XA[KS2024]-039-002/003), and informed consent was waived owing to the retrospective nature of the study.

### MALDI-TOF MS spectral analysis

MALDI-TOF MS identification was performed independently using two systems: the Bruker Microflex LT equipped with the MALDI Biotyper v9.0 database, and the Zybio EXS3000 system utilizing the MicroID v3.2 database, strictly following the manufacturers’ protocols. Spectra were acquired in linear positive ion mode (20 kV acceleration voltage, 2,000—20,000 Da mass range), with *Escherichia coli* DH5α serving as the mass calibrant.

For spectral feature extraction using the Bruker system, “summed spectra” were generated by averaging at least 20 single-shot mass spectra per strain after baseline correction and denoising. Main Spectra Profile (MSP) generation and mass peak calling (signal-to-noise ratio ≥ 3, peak width deviation ≤ 500 ppm) were conducted using MALDI Biotyper and FlexAnalysis 3.4 software. For analysis on the Zybio system, mass spectra were acquired in linear positive ion mode, with at least 30 separate sum spectra obtained for each strain. Isolate identification and spectral analysis were performed via the system’s integrated software using the built-in standard pattern matching approach, which automatically normalized and recalibrated the acquired spectra for high-confidence species-level identification. Preliminary candidate MS peaks were subsequently identified by comparing peak presence/absence and intensity differences across strains within this local dataset. Notably, to further evaluate the spectral correlations between *S. argenteus* and *S. aureus* isolates, hierarchical clustering analysis (HCA) and principal component analysis (PCA) were performed exclusively using the EXS3000 v3.7.4.12 software (Zybio Inc.) based on the spectral data acquired by the Zybio system, as previously described (Wang et al., 2023).

### Control strain selection and analysis

Briefly, the 21 *S. aureus* control strains were selected as follows: (1) they were randomly sampled from the 2,080 isolates to represent different years (2014–2025), specimen types (blood, sputum, etc.), and antimicrobial susceptibility profiles, comprising 14 methicillin-susceptible *S. aureus* (MSSA) and 7 methicillin-resistant *S. aureus* (MRSA); (2) the 2:1 MSSA-to-MRSA ratio approximates the general distribution in our hospital collection. MALDI-TOF MS dendrograms were constructed utilizing default parameters (distance metric: cosine similarity; linkage algorithm: complete linkage), and a correlation matrix was generated to assess spectral relationships among the isolates.

### Molecular identification

Genomic DNA was extracted using a phenol-chloroform method. PCR amplification of 16S rRNA (forward: 5′-AGAGTTTGATCATGGCTC-3′, reverse: 5′- TAGGGTTACCTTGTTACGACTT-3′) (Ng et al., 2009), *rpoB* (forward: 5′- AACCAATTCCGTATIGGTTT -3′, reverse: 5′-CCGTCCCAAGTCATGAAAC-3′) (Ng et al., 2009) and *NRPS* (forward: 5′- TTGARWCGACATTACCAGT-3′, reverse: 5′-ATWRCRTACATYTCRTTATC-3′) genes (Zhang et al., 2016) was performed, and amplicons were sequenced by Ruibio BioTech Co., Ltd., (Beijing, China). Sequences were aligned with reference genomes using BLAST^1^. PCR amplifications of the *blaZ* gene and Panton-Valentine leukocidin (PVL) gene were performed using the methods established by Martineau et al. (2000) and Lina et al. (1999).

### Antimicrobial susceptibility testing (AST)

Minimum inhibitory concentrations (MICs) were determined using a VITEK 2 Compact system (bioMérieux, Marcy-l’Étoile, France) with VITEK 2 GP67 cards, and further validated by the broth microdilution (BMD) method according to Clinical and Laboratory Standards Institute (CLSI) guidelines. Susceptibility results were interpreted using CLSI M100 guidelines (35th ed.) breakpoints, except for tigecycline, for which European Committee on Antimicrobial Susceptibility Testing (EUCAST) v15.0 breakpoints (≤ 0.5 μg/mL for susceptible) were used (The European Committee on Antimicrobial Susceptibility Testing, 2025; Clinical and Laboratory Standards Institute, 2025). Cefoxitin screening tests and D-tests were performed using the disk diffusion method as specified in CLSI M100 guidelines (35th ed.). *S. aureus* ATCC 29213 served as a quality control strain for all antimicrobial susceptibility tests.

### Biofilm formation assay

Biofilm formation was quantified using a polystyrene 96-well flat-bottom microtiter plate assay according to a previously described method (He et al., 2017) with slight modifications, with *S. aureus* ATCC 29213 serving as the positive control. Bacterial suspensions (1:100 dilution in tryptic soy broth [TSB] supplemented with 1% glucose) were added to each well in a 200 μL volume and incubated at 37 °C for 24 h. Adherent biofilms were stained with 0.1% crystal violet for 15 min, eluted with 95% ethanol for 10 min, and absorbance was measured at 590 nm (OD_590_), which is the standard detection wavelength for crystal violet staining. Biofilm formation was categorized as strong (OD_590_ > 4 × ODc), moderate (2 × ODc < OD_590_ ≤ 4 × ODc), weak (ODc < OD_590_ ≤ 2 × ODc), or non-biofilm-forming (OD_590_ ≤ ODc), where ODc is the mean OD of negative controls plus 3 times the standard deviation (SD) (ODc = mean OD + 3 × SD). All experiments were performed in triplicate with three independent biological replicates.

### Whole genome sequencing (WGS) and analysis

Complete genomes of *S. argenteus* isolates were sequenced using a hybrid short- and long-read approach. Short-read sequencing (150-bp paired-end) was performed on the DNBSEQ-T7 platform (MGI Tech, Shenzhen, China). Long reads were generated on the MGI CycloneSEQ nanopore sequencing platform using the CycloneSEQ Universal Library Prep Kit with a size selection threshold of > 8 kb. Raw DNBSEQ-T7 short reads were quality-trimmed using Trimmomatic (v0.36) to remove adapters and low-quality bases. The quality and metrics of the CycloneSEQ long reads—including read count, total bases, maximum length, N50, N90, and average read length—were assessed based on sequencing summary statistics. Hybrid *de novo* assembly was executed via Unicycler (v0.4.8) under default parameters to generate complete genome assemblies. Briefly, Unicycler leveraged the short-read assembly graphs produced by SPAdes (v3.14.0) and utilized the CycloneSEQ long reads to bridge contigs and resolve complex repetitive regions. The final assemblies were validated by mapping the sequencing reads back to the consensus genomes, followed by evaluation of GC content and read-depth distribution to screen for potential assembly artifacts, contamination, or uneven coverage. Protein-coding sequences, tRNAs, and rRNAs were predicted using GeneMarkS (v4.17), tRNAscan-SE (v2.0.4), and RNAmmer (v1.2), respectively (Chan et al., 2021). Antimicrobial resistance (AMR) determinants were annotated by NCBI blastp (v2.9.0+) searches against the Comprehensive Antibiotic Resistance Database (CARD v3.2.9) with an E-value cutoff of 1 × 10^–5^, retaining up to five target sequences for each query. Virulence factors were identified using DIAMOND blastp (v0.9.22) against the Virulence Factor Database (VFDB, 2024 update), with an E-value cutoff of 1 × 10^–5^, a sequence identity threshold of ≥ 80%, and a subject coverage of ≥ 90%.

To evaluate the genomic relatedness between the clinical isolates and reference strains, Average Nucleotide Identity (ANI) was calculated using the BLASTN algorithm (ANIb) via the pyani package (version 0.2.12). Three reference genomes representing the SAC were utilized for comparison: *S. argenteus* MSHR1132 (GenBank accession no. GCA_000236925.1), *S. aureus* subsp. *aureus* NCTC8325 (GenBank accession no. GCA_000013425.1), and *S. schweitzeri* NCTC13712 (GenBank accession no. GCA_900636685.1). Additionally, digital DNA-DNA hybridization (dDDH) values were determined using the Genome-to-Genome Distance Calculator (GGDC 3.0) online server^2^ based on Formula 2.

To ensure data quality, all publicly available *S. argenteus* genomes in NCBI GenBank as of December 2025 were evaluated based on the following inclusion criteria: (1) formal taxonomic classification as *S. argenteus*; (2) assembly status designated as complete genomes or high-quality draft assemblies; and (3) a minimum core genome coverage of ≥ 80% relative to the reference during alignment, which filtered out contaminated or heavily fragmented sequences. A total of 267 public genomes met these criteria (excluding the type strain). Together with our three clinical isolates, these genomes were analyzed using Snippy for variant calling and alignment against the reference genome *S. argenteus* MSHR1132 (GenBank accession no. GCA_000236925.1), which was selected as it represents the definitive, high-quality, completely assembled type strain for this species. A full-length core genome alignment, retaining all invariant and variant homologous sites shared across all isolates, was generated using snippy-core. To eliminate the confounding effects of homologous recombination, this full-length alignment was processed using Gubbins (v2.3.4) to identify and mask putative recombinant regions. Finally, a maximum-likelihood (ML) phylogenetic tree was constructed from the recombination-filtered, full-sequence alignment using IQ-TREE (v2.2.1). The optimal nucleotide substitution model was automatically selected using ModelFinder implemented in IQ-TREE, and node reliability was evaluated via 1,000 ultrafast bootstrap replicates.

### Statistical analysis

All data are presented as the mean ± standard error of the mean (SEM) from at least three independent experiments. Statistical analyses were performed using SPSS 26.0 software (IBM Corp., Armonk, NY, USA).

## Results

### Prevalence and clinical characteristics of *S. argenteus* isolates

In this study, we identified three *S. argenteus* strains (0.14% of 2,080 clinical samples) within our screening cohort, illustrating a low local prevalence in this clinical environment. The clinical metadata for the three isolates were as follows. BJS01 was recovered from a blood culture sample of an 89-year-old female patient presenting with community-acquired sepsis and multiple underlying comorbidities, including diabetes mellitus, hypertension, and cerebral infarction, which ultimately resulted in a fatal outcome; BJS02 was isolated from a sputum sample of a 70-year-old female patient diagnosed with hospital-acquired pneumonia; and BJS03 was obtained from a sputum sample of a 32-year-old male patient with pneumonia. Both patients infected with BJS02 and BJS03 achieved full clinical recovery and were successfully discharged.

### MALDI-TOF MS identification and spectral analysis

**FIGURE 2:**
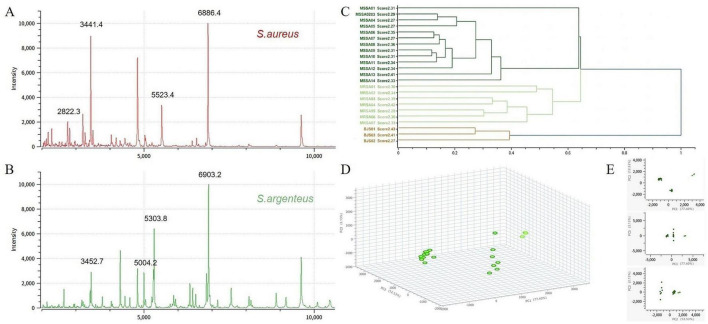
MALDI-TOF mass spectrometry profiles of *S. aureus* and *S. argenteus*, accompanied by hierarchical clustering analysis (HCA) and principal component analysis (PCA). **(A,B)** Unique mass peaks serving as candidate MS markers for *S. aureus* and *S. argenteus*. **(C)** HCA dendrogram confirming the separation of *S. argenteus* (*n = 3*, orange) from MSSA (*n = 14*, dark green) and MRSA (*n = 7*, light green). **(D,E)** 2D and 3D PCA plots visualizing the spatial distribution, clustering patterns, and intra-group heterogeneity of the isolates. The first three principal components account for 93.06% of the total variance (PC1 = 77.40%, PC2 = 12.53%, and PC3 = 3.13%). Dark green indicate *S. aureus* (with MSSA and MRSA clustering separately), while light green indicates *S. argenteus* (forming a distinct cluster). MALDI-TOF MS, matrix-assisted laser desorption/ionization-time of flight mass spectrometry; HCA, hierarchical clustering analysis; PCA, principal component analysis; MSSA, methicillin-susceptible *Staphylococcus aureus*; MRSA, methicillin-resistant *Staphylococcus aureus*.

MALDI-TOF MS (Zybio EXS3000) identified four preliminary candidate MS peaks each for *S. argenteus* (3452.7, 5004.2, 5303.8, and 6903.2 Da) and *S. aureus* (2822.3, 3441.4, 5523.4, and 6886.4 Da) (Figures 2A,B). These peaks were consistently detected across technical replicates, with high reproducibility (CV < 10% for peak intensity) serving as preliminary identification references within our single-laboratory framework.

HCA and PCA of spectral profiles clearly segregated *S. argenteus* from MSSA and MRSA into distinct clusters (Figure 2). HCA dendrogram of 24 strains resolved three distinct clusters matching phenotypic designations: 14 MSSA, 7 MRSA, and 3 *S. argenteus* strains, demonstrating clear proteomic separation among the isolates within the present dataset (Figure 2C). PCA revealed tight clustering of MSSA and *S. argenteus* isolates in 3D space, whereas MRSA isolates dispersed along the PC3 axis (which explained 3.13% of the variance; Figure 2D). With the first three principal components capturing a cumulative 93.06% of the total variance, the 2D score plot of PC1 versus PC2 (accounting for 77.40% and 12.53% of the variance, respectively) distinctly segregated all three groups (*S. argenteus*, MRSA, and MSSA), consistent with interspecies differences observed across analyses (Figure 2E).

### Molecular identification of isolates

Molecular sequencing confirmed 100% sequence identity with *S. argenteus* reference genomes (CP086574.1, AP024201.1, CP151186.1) in the *rpoB* and *NRPS* genes, while 16S rRNA genes sequencing failed to distinguish *S. argenteus* from *S. aureus* (sequence homology > 99.8%). The *rpoB* gene sequences generated and analyzed during the current study are available in the NCBI GenBank repository under accession numbers PX849481, PX849482, and PX849483.

### Antimicrobial susceptibility profiles

The three *S. argenteus* isolates were uniformly susceptible to oxacillin (MIC ≤ 0.25 μg/mL) and the majority of non-β-lactam classes tested (fluoroquinolones, aminoglycosides, macrolides, and glycopeptides). Regarding penicillin susceptibility, BJS01 and BJS03 were resistant (MIC ≥ 0.5 μg/mL), whereas BJS02 remained susceptible. Additionally, BJS03 displayed resistance to tetracycline (MIC ≥ 16 μg/mL) (Table 1). All results of quality control strain were within the acceptable range specified by CLSI M100 guidelines (35th ed.).

**TABLE 1:** *In vitro* antimicrobial susceptibility profiles of the three *S. argenteus* isolates.

Antimicrobial agent	BJS01 (μg/mL)	BJS02 (μg/mL)	BJS03 (μg/mL)	CLSI Breakpoints (μg/mL, S/I/R)
Penicillin G	≥ 0.5, R	0.06, S	≥ 0.5, R	0.12 / - / 0.25
Oxacillin	≤ 0.25, S	≤ 0.25, S	≤ 0.25, S	≤ 2 / -/ ≥ 4
Gentamicin	≤ 0.5, S	≤ 0.5, S	≤ 0.5, S	≤ 4 / 8 / ≥ 16
β-Lactamase	Positive	Negative	Positive	–
Rifampin	≤ 0.5, S	≤ 0.5, S	≤ 0.5, S	≤ 1 / 2 / ≥ 4
Moxifloxacin	≤ 0.25, S	≤ 0.25, S	≤ 0.25, S	≤ 0.5 / 1 / ≥ 2
Levofloxacin	≤ 0.12, S	0.25, S	0.25, S	≤ 1 / 2 / ≥ 4
Ciprofloxacin	≤ 0.5, S	≤ 0.5, S	≤ 0.5, S	≤ 1/ 2 / ≥ 4
Trimethoprim/sulfamethoxazole	≤ 0.5/9.5, S	≤ 0.5/9.5, S	≤ 0.5/9.5, S	≤ 2/38 / -/ ≥ 4/76
Clindamycin	≤ 0.25, S	≤ 0.25, S	≤ 0.25, S	≤ 0.5 / 1–2 / ≥ 4
Erythromycin	≤ 0.25, S	≤ 0.25, S	≤ 0.25, S	≤ 0.5 / 1–4 / ≥ 8
Inducible clindamycin resistance (D-test)	Negative	Negative	Negative	–
Nitrofurantoin	≤ 16, S	≤ 16, S	≤ 16, S	≤ 32 / 64 / ≥ 128
Linezolid	2, S	1, S	1, S	≤ 4 / - / ≥ 8
Vancomycin	1, S	1, S	1, S	≤ 2 / 4–8 / ≥ 16
Quinupristin/Dalfopristin	≤ 0.25, S	≤ 0.25, S	≤ 0.25, S	≤ 1/ 2 / ≥ 4
Tetracycline	≤ 1, S	≤ 1, S	≥ 16, R	≤ 4/ 8 / ≥ 16
Tigecycline ^a^	≤ 0.12, S	≤ 0.12, S	≤ 0.12, S	≤ 0.5 /-/ -
Cefoxitin Screen	Negative	Negative	Negative	–

^a^Tigecycline was interpreted according to EUCAST v15.0 due to the absence of CLSI criteria, while all other antibiotics were evaluated based on CLSI M100 guidelines (35th ed.). Breakpoints for *S. aureus* were applied to *S. argenteus*. MICs were determined by the broth microdilution method. “-”, No reference range or breakpoints. S, susceptible; I, intermediate; R, resistant; CLSI, Clinical and Laboratory Standards Institute; EUCAST, European Committee on Antimicrobial Susceptibility Testing.

### Biofilm formation capacity

**FIGURE 3:**
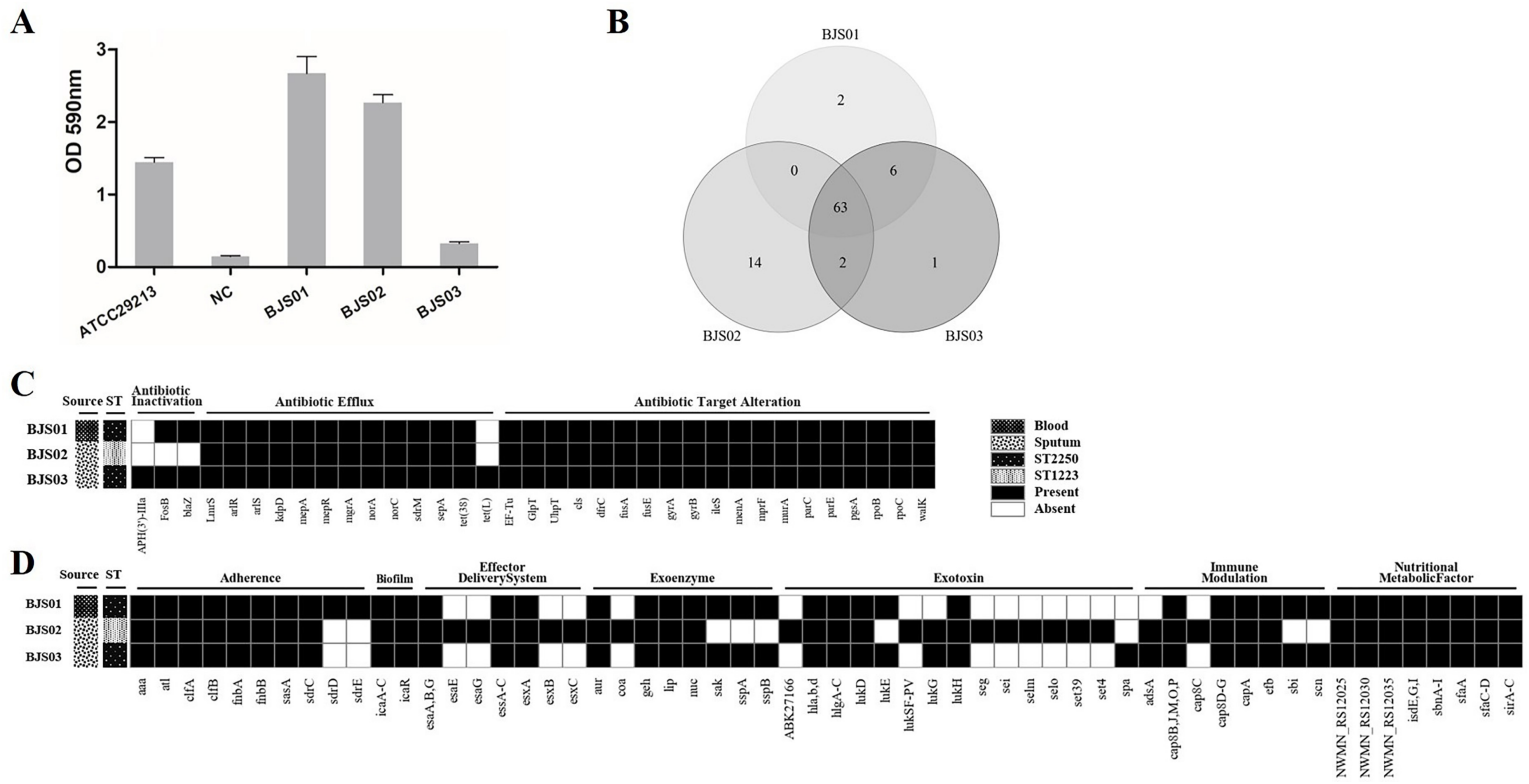
Genomic and phenotypic characterization of virulence and antimicrobial resistance profiles of three *S. argenteus* isolates. **(A)** Biofilm formation capability of the three strains. Compared with the control strain (*S. aureus* ATCC 29213), BJS01 and BJS02 exhibited stronger biofilm formation capability. All experiments were performed in triplicate with three independent biological replicates. **(B)** Venn diagram showing the number of shared (*n* = 63) and strain-specific virulence genes among the three strains. **(C)** Presence-absence heatmap of antimicrobial resistance genes annotated using the CARD database based on WGS. **(D)** Presence-absence heatmap of virulence factor genes annotated via the VFDB database. For both heatmaps **(C,D),** black indicates the presence of a gene, while white indicates its absence. WGS, whole genome sequencing; CARD, Comprehensive Antibiotic Resistance Database; VFDB, Virulence Factor Database.

The three *S. argenteus* isolates exhibited variable biofilm-forming capacities. BJS01 and BJS02 formed strong biofilms with OD_590_ values of 2.67 ± 0.40 and 2.27 ± 0.19, respectively, both of which were numerically higher than that of the positive control strain *S. aureus* ATCC 29213 (OD_590_ = 1.44 ± 0.11). In contrast, BJS03 formed a weak biofilm (OD_590_ = 0.32 ± 0.04) (Figure 3A). Although the *n* = 3 biological replicates were sufficient for this exploratory analysis, larger-scale cohorts are required to draw confirmatory conclusions.

### Resistome characteristics and virulence profiling

A Venn diagram analysis of virulence genes identified a total of 88 unique genes across the three isolates. Among these, 63 genes constituted the shared core virulome, while 2, 14, and 1 were strain-specific for BJS01, BJS02, and BJS03, respectively (Figure 3B). In addition to virulence determinants, the genomic profiles of antimicrobial resistance were also evaluated.

All isolates harbored efflux pump genes (e.g., *norA/B/C* and *lmrS*) and regulatory elements (e.g., *mgrA*, *mepr*, *arlS*, and *arlR*); however, these elements did not confer *in vitro* resistance under standard laboratory testing conditions (Figure 3C and Table 1). WGS revealed a conserved mutation within the fluoroquinolone resistance-determining regions (QRDRs) of *gyrA* (Ser84Leu) across all isolates. Additionally, no *mecA* or *mecC* genes were detected, which perfectly aligned with their oxacillin-susceptible phenotypes.

Genomic analysis further demonstrated a highly conserved core virulome among all *S. argenteus* isolates, encompassing adhesion and biofilm-related genes (*clfA*, *clfB*, *fnbA*, *fnbB*, and *icaA–C*), hemolysins (*hla*, *hlb*, *hld*, and *hlgA–C*), leukotoxins (*lukD* and *lukE*), immune evasion factors (*spa* and *sbi*), and the capsular polysaccharide biosynthesis gene cap8 (Figure 3D). Notably, BJS02 uniquely carried Panton-Valentine leukocidin (*lukS/F-PV*) and enterotoxin genes (*seg* and *sei*), which serve as genomic markers of heightened virulence potential. None of the strains harbored the toxic shock syndrome toxin gene (*tst*) or exfoliative toxin genes (*eta* and *etb*).

### Whole-genome assembly statistics and species assignment

To establish taxonomic certainty in accordance with contemporary standards, whole-genome metrics were extracted. The assemblies exhibited an average size of 2.81 Mb, an N50 value of > 150 kb, and a GC content of approximately 32.5%. High completeness ( > 99.0%) and low contamination ( < 0.5%) metrics were confirmed across all three genomes. Comprehensive assembly statistics—including contig counts, sequencing depth, total CDS, and rRNA/tRNA counts—are systematically summarized in Supplementary Table 1.

**FIGURE 4:**
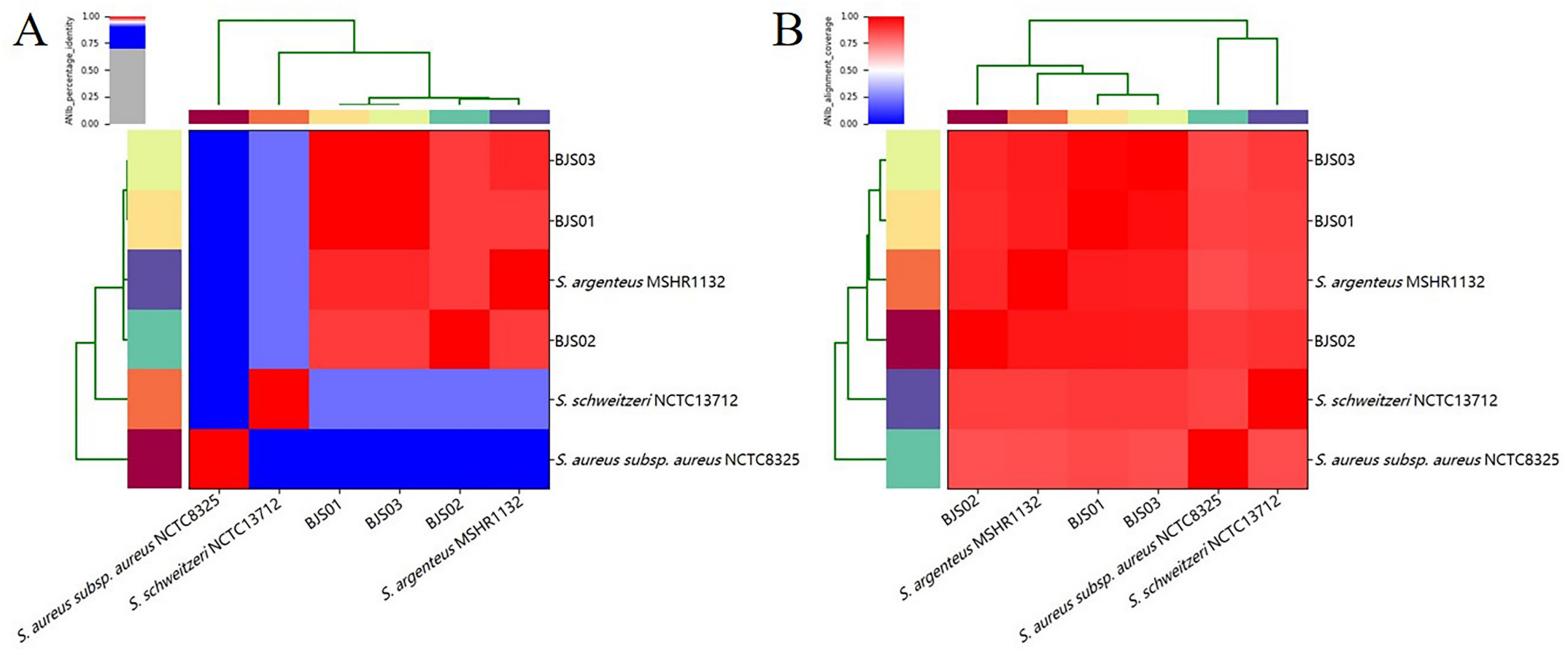
Hierarchical clustering heatmaps of Average Nucleotide Identity (ANIb) and genomic alignment coverage among the three clinical isolates and reference strains. **(A)** Inter-strain ANIb identities are 98.66% (BJS01 vs. BJS02), 99.91% (BJS01 vs. BJS03), and 98.67% (BJS02 vs. BJS03). ANIb values for BJS01, BJS02, and BJS03 against reference strains are: *S. argenteus* MSHR1132 (98.89%, 98.71%, and 98.90%, respectively); *S. aureus* subsp. *aureus* NCTC8325 (87.67%, 87.53%, and 87.54%, respectively); and *S. schweitzeri* NCTC13712 (92.03%, 92.09%, and 92.03%, respectively). **(B)** The pairwise alignment coverages among the three isolates range from 92.13% to 98.58%, with a minimum value of 83.87% observed when compared against all reference genomes.

Genomic taxonomic analysis confirmed that the three clinical isolates belong to *S. argenteus*. Specifically, strains BJS01, BJS02, and BJS03 exhibited high ANIb ( > 98.67%) and dDDH ( > 88.50%) values when compared with *S. argenteus* MSHR1132, well above the standard species definition thresholds (95% for ANI and 70% for dDDH). In contrast, their ANIb values were significantly lower when compared against *S. aureus* subsp. *aureus* NCTC8325 (ranging from 87.52% to 87.67%) and *S. schweitzeri* NCTC13712 (ranging from 91.98% to 92.13%) (Figure 4). Detailed pairwise ANI, coverage, and dDDH values are provided in Supplementary Table 2.

### MLST genotyping and phylogenetic analysis

**FIGURE 5:**
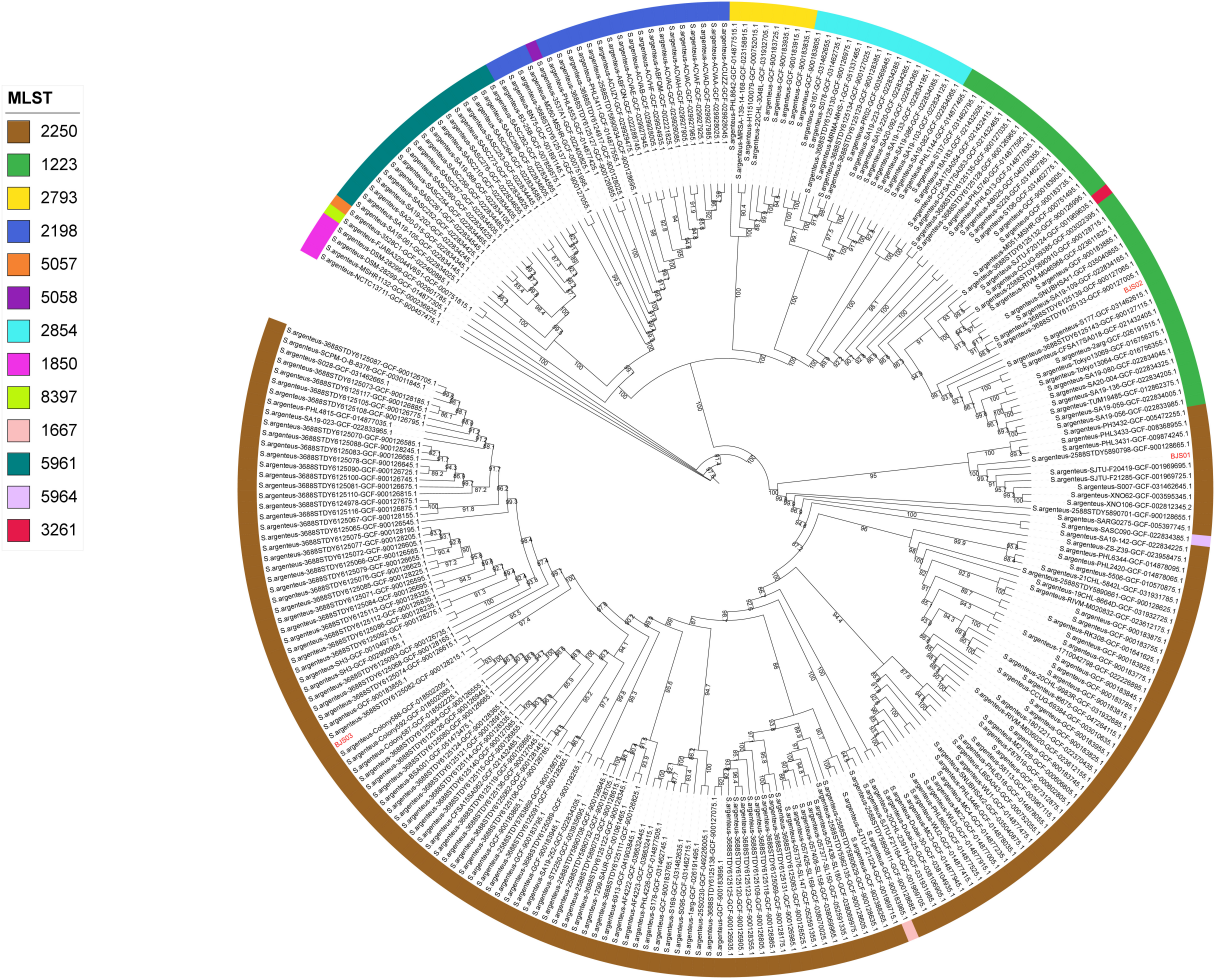
Maximum-likelihood phylogenetic tree of 270 *S. argenteus* genomes. The tree was constructed from a recombination-filtered, full-length core genome alignment comprising 267 public genomes retrieved from NCBI GenBank and the three local isolates from this study, using the type strain MSHR1132 as the reference. Major sequence types (STs), predominantly ST2250 and ST1223, are differentiated by the colored outer rings. The three clinical isolates sequenced in this study (BJS01, BJS02, and BJS03) are explicitly highlighted in red text. The tree is midpoint-rooted. Node reliability was evaluated via 1,000 bootstrap replicates, with support values >85% displayed at the branches, demonstrating robust lineage and clade stability. The scale bar represents the number of nucleotide substitutions per site.

MLST analysis revealed that the three local isolates belonged to two globally distributed lineages: ST2250 (BJS01 and BJS03) and ST1223 (BJS02). The raw sequencing reads of these three clinical isolates have been deposited in the NCBI GenBank database under the BioProject accession number PRJNA1440927. Core-genome phylogenetic reconstruction demonstrated that the 270 *S. argenteus* genomes were predominantly composed of ST2250 and ST1223, interspersed with diverse sequence types such as ST2793 and ST2198 (Figure 5). On the ML tree, our clinical isolates clustered robustly within their respective ST-specific clades. Notably, although BJS01 and BJS03 were both assigned to the ST2250 lineage, a pairwise distance of 228 SNPs was observed between them. This genomic divergence effectively rules out a recent, direct point-source intra-hospital transmission, suggesting instead independent introduction events or ongoing microevolution of the ST2250 background population in this region. In contrast, inter-ST genomic divergence aligned with macro-evolutionary divisions; BJS02 (ST1223) exhibited 15,969 and 16,018 SNP differences from BJS01 and BJS03, respectively. Across the entire dataset, the pairwise SNP distances among all 270 genomes ranged widely from 1 to 16,471 SNPs (with a median of 12,840 SNPs). Detailed geographic metadata, lineage representations, and genome accession numbers are compiled in Supplementary Table 3, and the complete pairwise SNP distance matrix is provided in Supplementary Table 4.

## Discussion

In this exploratory study, we report preliminary observations on three *S. argenteus* isolates from a single tertiary hospital in Beijing. Our findings provide three preliminary insights that advance our understanding of *S. argenteus* in low-prevalence, temperate clinical settings. First, we characterized its prevalence within a large clinical cohort, noting an extremely low isolation rate (0.14%), despite the presence of globally distributed lineages (ST2250 and ST1223) (Moradigaravand et al., 2017; Aung et al., 2019). Second, we identified four candidate MALDI-TOF MS spectral peaks (3452.7, 5004.2, 5303.8, and 6903.2 Da) with the potential to distinguish these strains from *S. aureus*. Third, phenotypic and genomic analyses demonstrated that strong biofilm-forming capacity and a conserved virulome coexist within these oxacillin-susceptible isolates, reflecting their clinical similarity to *S. aureus* (Goswami et al., 2021).

Our study integrated MS peak analysis with WGS validation to screen potential biomarkers, addressing the critical diagnostic challenge of distinguishing *S. argenteus* from *S. aureus* in routine clinical settings. The application of these candidate spectral markers may reduce identification time compared to the 24–48 h required for WGS (Becker et al., 2019). Specifically, the candidate MS peaks at *m/z* 5004.2 and 6903.2 identified in our study are consistent with previously reported peaks at *m/z* 5005.7 and 6905.5, respectively (Chen et al., 2018). Additionally, two other distinct candidate peaks observed in this study might be associated with the diversity of sequence types (STs) among the strains. However, these markers were identified within a single-laboratory setting using a limited sample size of *S. argenteus* isolates, and they lack external sensitivity or receiver operating characteristic (ROC) assessments. Therefore, further validation in larger, multicenter clinical blinded cohorts and across different mass spectrometry platforms is essential to confirm their generalized diagnostic value.

Additionally, although genomic analysis revealed the presence of *gyrA* mutations and efflux pump genes, all isolates remained phenotypically susceptible to fluoroquinolones. This demonstrates that a single *gyrA* Ser84Leu mutation typically confers only a modest increase in the MIC and is insufficient to produce clinical-level fluoroquinolone resistance without additional mutations in *grlA*/*parC* (Schmitz et al., 1998; Hooper, 2002; Kim et al., 2025). Future studies will focus on clarifying the underlying resistance mechanisms to fully validate this hypothesis.

Genomic analysis further revealed a highly conserved core virulome across all isolates—comprising adhesins, hemolysins, and immune evasion factors—which substantially overlapped with the virulence profile of *S. aureus* (Johansson et al., 2019). Notably, strain BJS02 (ST1223) uniquely harbored Panton-Valentine leukocidin (PVL) genes (*lukS/F-PV*) and enterotoxin genes (*seg*, *sei*). This represents a rare detection of PVL-positive *S. argenteus* within this geographic region, underscoring the pathogenic potential of globally circulating clones in non-endemic areas. However, it is important to note that our virulence gene analysis is based solely on genomic presence/absence profiles. The presence of virulence genes does not necessarily indicate active expression, functional pathogenicity, or phenotypic virulence; thus, *in vivo* infection models are required to establish true pathogenic potential.

Beyond genotypic virulence profiles, phenotypic traits such as biofilm-forming capacity are commonly linked to bacterial pathogenicity. However, it must be noted that the intra-species variations in biofilm robustness observed in our crystal violet assays were not validated at the molecular or structural levels. In this study, we did not investigate *ica* operon expression, *agr*/*sarA* regulation, *RNAIII* levels, or extracellular polysaccharide production, nor did we perform confocal or scanning electron microscopy. Therefore, while we hypothesize that the observed phenotypic variation may be associated with underlying gene regulation or expression efficiency, this remains strictly speculative and warrants comprehensive transcriptional profiling in future studies.

In this study, strain BJS01 exhibited the most robust biofilm-forming ability and was isolated from the patient with the poorest clinical outcome, suggesting a potential correlation between biofilm robustness and disease severity. However, this clinical correlation was confounded by the patient’s multiple severe comorbidities. Interestingly, although strain BJS02 also demonstrated strong biofilm-forming capacity (alongside its unique toxin profile), it was associated with a favorable clinical prognosis, similar to the weak biofilm-producing strain BJS03. The lack of a consistent linear correlation across all three isolates, particularly the genotype-phenotype discordance observed in BJS02, underscores the profound impact of host-related risk factors. Consequently, the precise relationship between biofilm-forming capacity and the intrinsic virulence of these *S. argenteus* isolates warrants further systematic validation using defined cellular or animal infection models.

We performed phylogenetic and molecular epidemiological analyses of 270 *S. argenteus* genomes—comprising 267 public reference sequences from NCBI and 3 newly sequenced isolates—and confirmed ST2250 and ST1223 as the dominant globally circulating clonal lineages of this emerging opportunistic pathogen. These two lineages exhibit substantial genome-wide genetic divergence (Aung et al., 2025; Zhou et al., 2026). Notably, we detected significant intra-lineage genetic diversity within ST2250: our two ST2250 isolates clustered in distinct subclades despite identical traditional MLST profiles, suggesting that high-resolution phylogenomics inherently provides greater discriminatory power than standard MLST in resolving fine-scale genetic variations in *S. argenteus*.

The two dominant lineages display distinct epidemiological and genomic traits: ST1223, a globally distributed lineage, is associated with foodborne staphylococcal intoxication via enterotoxin/superantigen genes (with low PVL prevalence), whereas ST2250, the more prevalent lineage, harbors a richer repertoire of antimicrobial resistance genes and is linked to both community- and hospital-acquired infections (Aung et al., 2021; Goswami et al., 2021). These findings provide exploratory insights into the evolutionary relationships of common *S. argenteus* lineages and highlight the value of core-genome SNP-based high-resolution analysis for detailed molecular surveillance and precise source tracking of this pathogen.

Several limitations of this study should be acknowledged. First, although our findings are derived from a screening cohort of 2,080 SAC isolates, the naturally low prevalence of *S. argenteus* in our local cohort yielded only three positive strains. Consequently, our downstream characterizations—including candidate MALDI-TOF MS spectral markers, epidemiological features, and virulence phenotypes—are inherently preliminary. While this low recovery accurately reflects the regional epidemiology rather than an inadequate sampling effort, these specific findings require validation in broader, multicenter cohorts. Second, several methodological constraints exist, including single-laboratory mass peak identification without multi-platform validation, as well as a lack of functional experiments and confirmation of resistant mechanisms (such as RT-qPCR, *RNAIII* expression assays, or scanning electron microscopy for biofilms). Accordingly, future studies incorporating multicenter surveillance, resistant mechanisms studies and functional genomic analyses are warranted to clarify the clinical significance of *S. argenteus*. Third, due to logistical and biosecurity constraints in obtaining international live reference type strains of *S. argenteus* (e.g., *S. argenteus* MSHR1132) during our retrospective study window, these standard strains were not analyzed under identical *in vitro* laboratory conditions for MALDI-TOF MS spectral patterns or phenotypic biofilm assays. To compensate for this, high-resolution *in silico* genomic comparisons (ANI and dDDH) against these fully characterized global type strains were utilized to ensure rigorous taxonomic and genomic alignment.

## Conclusion

This exploratory study provides an integrated genomic and phenotypic characterization of three clinical *S. argenteus* isolates from a tertiary hospital in Beijing, elucidating their microbiological traits, virulence potential, and antimicrobial resistance profiles. We identified four preliminary candidate MALDI-TOF MS spectral peaks within our local dataset that could serve as diagnostic biomarkers for future, large-scale multicenter validation. Furthermore, the detection of a strain carrying PVL and enterotoxin genes, alongside its strong biofilm-forming capacity, suggests a notable virulence potential and heterogeneity among these local isolates. Overall, these preliminary findings underscore the clinical significance of *S. argenteus* as an emerging pathogen, emphasizing the urgent need for enhanced molecular surveillance and further functional research to provide a framework for future large-scale, multicenter surveillance.

## Data Availability

The datasets presented in this study can be found in online repositories. The names of the repository/repositories and accession number(s) can be found below: https://www.ncbi.nlm.nih.gov/, PRJNA1440927.

## Footnotes

1
https://blast.ncbi.nlm.nih.gov/Blast.cgi

2
https://ggdc.dsmz.de

## Supplementary material

Supplementary Table 2 Comprehensive pairwise genomic alignment parameters based on ANIb analysis and digital DNA-DNA hybridization (dDDH) values among the studied strains.

Supplementary Table 3 Geographic metadata and NCBI accession numbers of the public reference genomes utilized in this study.

Supplementary Table 4 Pairwise core-genome SNP distances among the local *S. argenteus* isolates.
