# Supplementary material for: Clinical and genomic characterization of Staphylococcus argenteus isolates from a tertiary hospital in Beijing
Source: Front Microbiol. 2026 Jul 28;17:1876812. doi: 10.3389/fmicb.2026.1876812 (PMC13457392; doi:10.3389/fmicb.2026.1876812)
Supplement: Supplementary file 1 [file Table_1.docx]

**Supplementary Table S1 Genomic assembly statistics of the three *S. argenteus***

| **Metric** | **BJS01** | **BJS02** | **BJS03** |
| --- | --- | --- | --- |
| Assembly size (bp) | 2,816,820 | 2,695,967 | 2,788,149 |
| Number of contigs | 2 | 1 | 2 |
| N50 (bp) | 2,796,095 | 2,695,967 | 2,759,051 |
| L50 | 1 | 1 | 1 |
| GC content (%) | 32.42 | 32.39 | 32.39 |
| Sequencing depth (x) | 371.25 | 424.21 | 410.08 |
| Completeness (%) | 100 | 99.99 | 100 |
| Contamination (%) | 0.25 | 1.87 | 0.17 |
| Number of CDS | 2668 | 2597 | 2633 |
| Number of rRNA genes | 19 | 19 | 19 |
| Number of tRNA genes | 59 | 59 | 59 |
| Accession number | SAMN61026977 | SAMN61026978 | SAMMN61026979 |

Abbreviations: bp, base pairs; N50, minimum contig length covering 50% of the genome; L50, minimum number of contigs covering 50% of the genome; CDS, Coding DNA Sequence; rRNA, ribosomal RNA; tRNA, transfer RNA.
